# Labelling of live cells using fluorescent aptamers: binding reversal with DNA nucleases

**DOI:** 10.1186/1477-3155-8-8

**Published:** 2010-04-13

**Authors:** Hideyuki Terazono, Yu Anzai, Mikhail Soloviev, Kenji Yasuda

**Affiliations:** 1 Kanagawa Academy of Science and Technology, KSP East 310, 3-2-1 Sakado, Takatsu, Kawasaki, Kanagawa 213-0012, Japan; 2 School of Biological Sciences, Royal Holloway, University of London, Egham, Surrey TW20 0EX, UK; 3 Department of Biomedical Information, Division of Biosystems, Institute of Biomaterials and Bioengineering, Tokyo Medical and Dental University, 2-3-10 Kanda-Surugadai, Chiyoda, Tokyo 101-0062, Japan

## Abstract

A reversible cell labelling method has been developed for non-destructive and non-invasive cell labelling and purification. Our method uses high affinity single strand DNA (ssDNA) aptamers against surface exposed target molecules on cells. The aptamers are subsequently removed from the cell surface using DNase nuclease treatment. We exemplified our method by labelling human acute lymphoblastic leukemia cells with Qdot-ssDNA aptamers, and restoring them to the label-free condition by treatment with Benzonase. Binding of the fluorescent-aptamers to the cells was evaluated by measuring fluorescence intensity and was further confirmed using flow cytometry. Removal of the aptamers can be achieved in ~10 min by the DNase nuclease digestion. Incubation of cells with aptamers or with the nucleases results in no apparent damage to the cells and does not affect their growth rates. The latter were equivalent to the rates measured for the untreated cells. Our method provides an alternative to traditional antibody-based techniques and could be especially suitable for non-invasive reversible cell labelling and cell separations where maintaining native cell activity is needed.

## Background

Separation of uniform phenotype cells whilst maintaining their native states is important both for post genome cell based studies and practical applications of those cells for regenerative medicine. A number of cell separation methods have been reported to date, such as density-gradient centrifugation, antibiotic screening and fluorescence-activated cell sorting (FACS). The latter gained significant popularity because of its simplicity and throughput. One of the problems of the FACS and other antibody-based labelling and separation techniques is that strong, often nearly irreversible conjugation between antibody and antigen molecules on cell surface may affect the function of the targeted cells and their interaction efficacy with other cells [1].

As an alternative to antibodies, we developed nucleotide based probes capable of binding target cell antigens [2]. Aptamers are single-strand DNAs (ssDNA), RNAs or modified nucleic acids. Systematic Evolution of Ligands by Exponential Enrichment (SELEX) procedures for molecular evolution and aptamer engineering have been reported previously [3,4]. The development of ssDNA aptamers for specific binding to live cells (Cell-SELEX) expanded their potential for use in cell separation and labelling [5-7]. However, the problem of removal of aptamers from the target cells and restoring cells to their original unlabelled state have not been addressed yet. Here we describe a method for reversible labelling of the cells with fluorescently tagged aptamers.

## Materials and methods

### Cell Culture

Human acute lymphoblastic leukemia cells, CCRF-CEM (Dainippon Sumitomo Pharma, Osaka, Japan), were cultured at 37°C under a 5% CO_2 _atmosphere in RPMI 1640 medium containing 10% heat-inactivated FBS, 100 IU/ml penicillin, and 100 μg/ml streptomycin (Invitrogen, Carlsbad, CA, USA).

### Target Aptamer and Negative Control Aptamer

5'-Biotin-labeled ssDNA was used as the target DNA aptamers for specific CCRF-CEM cell labelling, and random sequence ssDNA consisting of the same length and base composition as the target DNA aptamers were used as negative control DNA aptamers [6]. Sequence of recognition area of specific DNA aptamer was 5'-TTT AAA ATA CCA GCT TAT TCA ATT AGT CAC ACT TAG AGT TCT AGC TGC TGC GCC GCC GGG AAA ATA CTG TAC GGA TAG ATA GTA AGT GCA ATC T-3', and that of negative control ssDNA was 5'-TTT AAA GGT CAT AGT AAT ATG AGG TAA TAC AAG CAA TCG ACT AGG ACC AGC CTG TTA CGA CTA ATA TCG GCC TGC ACA TGG TGA TCT TCT CAT T-3'. Both ssDNAs were synthesized by Sigma Genosis (Hokkaido, Japan).

### Qdot-aptamer Labeling

**Figure 1:**
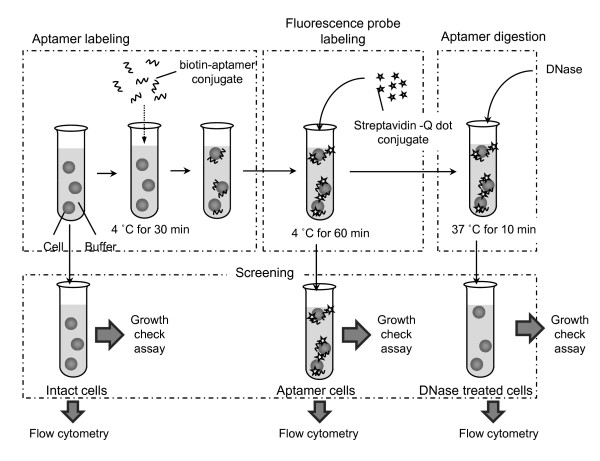
**An overview of the reversible aptamer labelling of live cells**. Intact cells are labelled with biotin-aptamer conjugate, followed by streptavidin-Qdot conjugates. Next, the Qdot-labelled aptamer cells are incubated with nuclease (DNase treated cells), this removes aptamers from the cells. The fluorescence intensities of intact cells, aptamer-labelled cells and DNase treated cells are compared using flow cytometery; possible cell damage is evaluated by measuring cell growth rates.

Figure 1 shows the procedure of cell labelling with 5'-biotin-labeled ssDNA aptamers followed by nuclease digestion. First, the cultured CCRF-CEM cells (intact cells) were washed three times with culture medium (RPMI 1640 medium containing 10% heat-inactivated FBS, 100 IU/ml penicillin, and 100 μg/ml streptomycin), and then incubated with the aptamer (4 μM) at 4°C for 30 min. After the incubation, the cells were washed three times with the cold culture medium to eliminate unbound aptamers.

Next, the 5'-Biotin of the ssDNA aptamers bound to the cells were labelled by addition of 0.1 μM Qdot-streptavidin conjugate (Qdot 525: emission maximum near 525 nm, Invitrogen Corp., Carlsbad, CA, USA). After 60 min incubation at 4°C, the cells were washed three times with the cold culture medium to remove excess free Qdot. These Qdot-aptamer labelled cells (aptamer cells) were then characterised by fow cytometry. To confirm the specificity of aptamer binding, both the target-specific aptamers and the negative control aptamers (irrelevant sequences) were used.

### Flow cytometry

Fluorescence measurements were made using an LSR II flow cytometer (Becton Dickinson, CA, USA). Qdot fluorescence of samples initially containing 10^6 ^cells in flow cytometry buffer (Dulbecco's PBS containing 4.5 g/L Glucose, 5 mM MgCl_2 _and 1% FBS) were measured by counting 30,000 events.

#### Nuclease Digestion

To remove the attached Qdot-aptamer from cells, the aptamer cells were incubated in the pre-incubation culture medium containing 0.4 Unit/μL Benzonase nuclease (Novagen, Darmstadt, Germany) at 37°C for 10 min. After the incubation, the cells were washed three times with the cold culture medium, and were characterised by flow cytometry as described above.

### Growth assay

To evaluate the invasiveness of nuclease digestion, the growth rates of the three kinds of aptamer-treated cells were measured after Benzonase nuclease treatment: cells treated with target-specific aptamer, with the negative control aptamer treatment, and those with no aptamer treatment. Aptamer attachment and Benzonase nuclease treatment proceeded as described above, and then cultured in a medium for six days. The same number of non-treated intact cells was also cultured as control. In each sample, the number of cells collected by centrifugation was counted using a Burker-Turk counting chamber. The growth rate represented the cell number normalized by those as 100% when growth-measurement started (day 0).

### Results and Discussion

**Figure 2:**
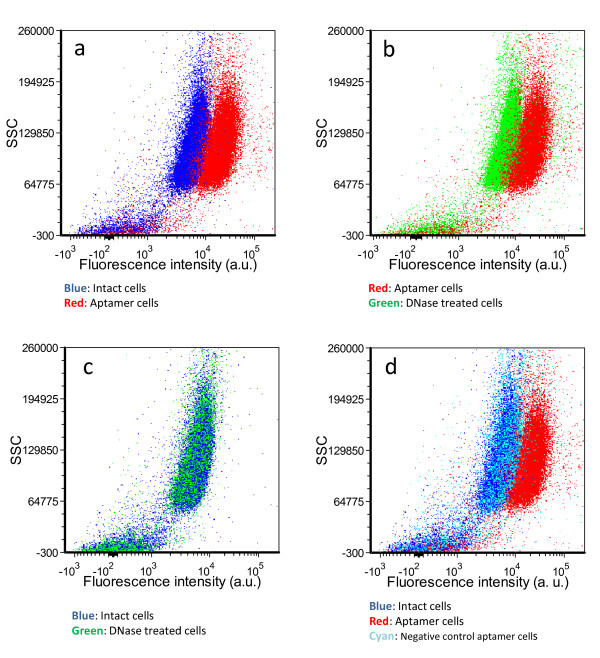
**Fluorescence intensity of the cells labelled with Qdot-aptamer and treated with nuclease**. Fluorescence changes following Qdot-aptamer labelling of intact cells (blue: intact cells, red: aptamer labelled cells) (a). Fluorescence changes following DNase treatment of aptamer cells (red: aptamer labelled cells, green: DNase treated cells) (b). Comparison of unlabelled cells (blue) and DNase treated cells (green) (c). Comparison between unlabelled cells (blue), cells incubated with an irrelevant aptamer (cyan) and cells labelled with cell-specific aptamer (red) (d). Vertical axes show Side Scatter (SSC) values, and horizontal axes show fluorescence intensity measured at 525 nm.

To this end we have developed and evaluated the procedure of non-destructive and reversible fluorescent labelling of live cells using DNA-aptamers (see Figure 1). First of all, we examined the labelling efficiency of the ssDNA target aptamers on intact cells using flow cytometry. Figure 2(a) shows an increase in fluorescence intensity of intact cells by over a hundred fold following the Qdot-aptamer treatment. Subsequently, we removed the attached DNA aptamers from cells using DNase nuclease as shown in Figure 1. We then examined the effect of DNase nuclease treatment on the Qdot-aptamer tagged cells. Figure 2(b) shows the decrease of fluorescence emission intensity of cells following the nuclease treatment. To confirm the efficiency of aptamer removal, we compared the fluorescence distributions of intact cells and DNase nuclease treated Qdot-aptamer cells. As shown in Figure 2(c), the distributions of the intact cells and the DNase nuclease treated cells overlapped each other. The results indicate that only the target-specific aptamer was attached to the cells (i.e., the negative control aptamer did not interact with target cells), and that the removal of Qdot-aptamer from the cells by DNase nuclease treatment allowed recovery of the initial distribution of intact cells. We also checked the interaction of non-specific negative control DNA aptamer with the intact cells. Figure 2(d) shows that the distribution of fluorescent intensities of Qdot-labelled negative control aptamer treated cells overlapped with that of the intact untreated cells, indicating lack of interaction and absence of any noticeable effects on the treated cells.

**Figure 3:**
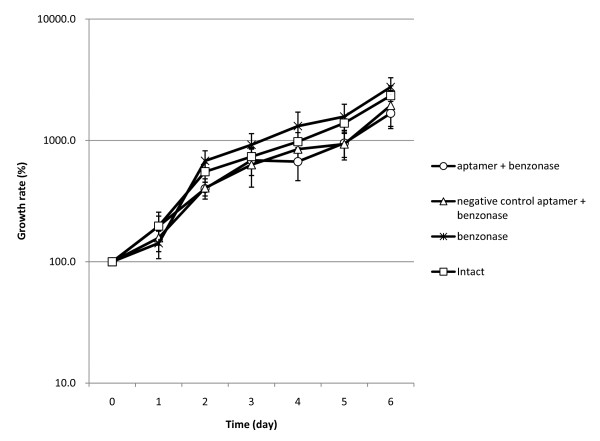
**Growth rates measured for the cells undergone aptamer labelling and its reversal by DNase treatment**. The growth rate (vertical axis) shows an increase in cell numbers normalized to the initial cell numbers at the start of cultivation time (i.e., day 0). Growth rates for the cells labelled with cell-specific aptamer and undergone subsequent DNase treatment (open circles). Growth rates for the cells treated with an irrelevant aptamer (open triangles); unlabelled cells treated with Benzonase (cross); control cells - unlabelled and untreated (open squares) (N = 6).

To evaluate possible damage to cells during labelling or nuclease digestion procedures we measured and compared the growth rates of intact and treated cells. Figure 3 shows the growth curves of the three types of nuclease-treated cells (cells incubated with the target-specific aptamer, with an irrelevant aptamer, or cells without an aptamer) and the non-treated intact cells as control. For five days, the growth rates of all three types of treated cells were almost the same as that of the non-treated intact cells. This result indicates that the aptamer labelling and DNase treatment have no apparent effect on the cells growth rates.

The conventional SELEX method is aimed at the development of DNA/RNA based drug-like molecules, and therefore aptamers need to be resistant to nuclease activity under physiological conditions, e.g. to plasma 3'-exonucleases [8,9]. Some of the previously reported aptamers acquired stability to 3'-exonucleases from human plasma [10,11]. The focus of this project was on the development of digestible aptamers, and their removal from the target cells by conventional DNases. Our approach is uniquely applicable to nucleic acid aptamers only, because live cells do not normally have DNAs associated with the cell surface, therefore nuclease treatment is not expected to elicit any functional effect. Traditional antibody-based approached to cell labelling would not allow similar treatment (protease digestion in the case of protein aptamers or antibodies), because this would strip the cells of many functionally important proteins exposed on the cell surface. In our experiment, we used Benzonase nuclease, an endonuclease with the ability to cleave the phosphodiester bonds at many digestion sites, and confirmed restoring the cells to a label-free condition.

In conclusion, we developed a reversible cell-labelling procedure in which live cells are labelled with fluorescent DNA aptamers, followed by their removal with nuclease treatment. Our labelling procedure produces no noticeable damage to the labelled cells. Our method is advantageous over traditional antibody labelling because it allows non-invasive cell separation and reversible cell labelling. It has a great promise for a variety of applications ranging from cell-based research to regenerative medicine.

## Authors' contributions

HT carried out whole experiments and participated in the design of the study and contributed to the drafting of the manuscript. YA carried out aptamer quality check and cell cultivation experiments. MS participated in the design of the study and the interpretation of results, and contributed to the drafting of the manuscript. KY conceived of the study, participated in its design and coordination and drafted the manuscript. All authors read and approved the final manuscript.

## Competing interests

The authors declare that they have no competing interests.
